# Supplementary material for: Tau forms synaptic nano-biomolecular condensates controlling the dynamic clustering of recycling synaptic vesicles
Source: Nat Commun. 2023 Nov 10;14:7277. doi: 10.1038/s41467-023-43130-4 (PMC10638352; doi:10.1038/s41467-023-43130-4)
Supplement: Supplementary file 1 — Supplementary Information [file 41467_2023_43130_MOESM1_ESM.pdf]

Supplementary Information

Supplementary Fig. S1

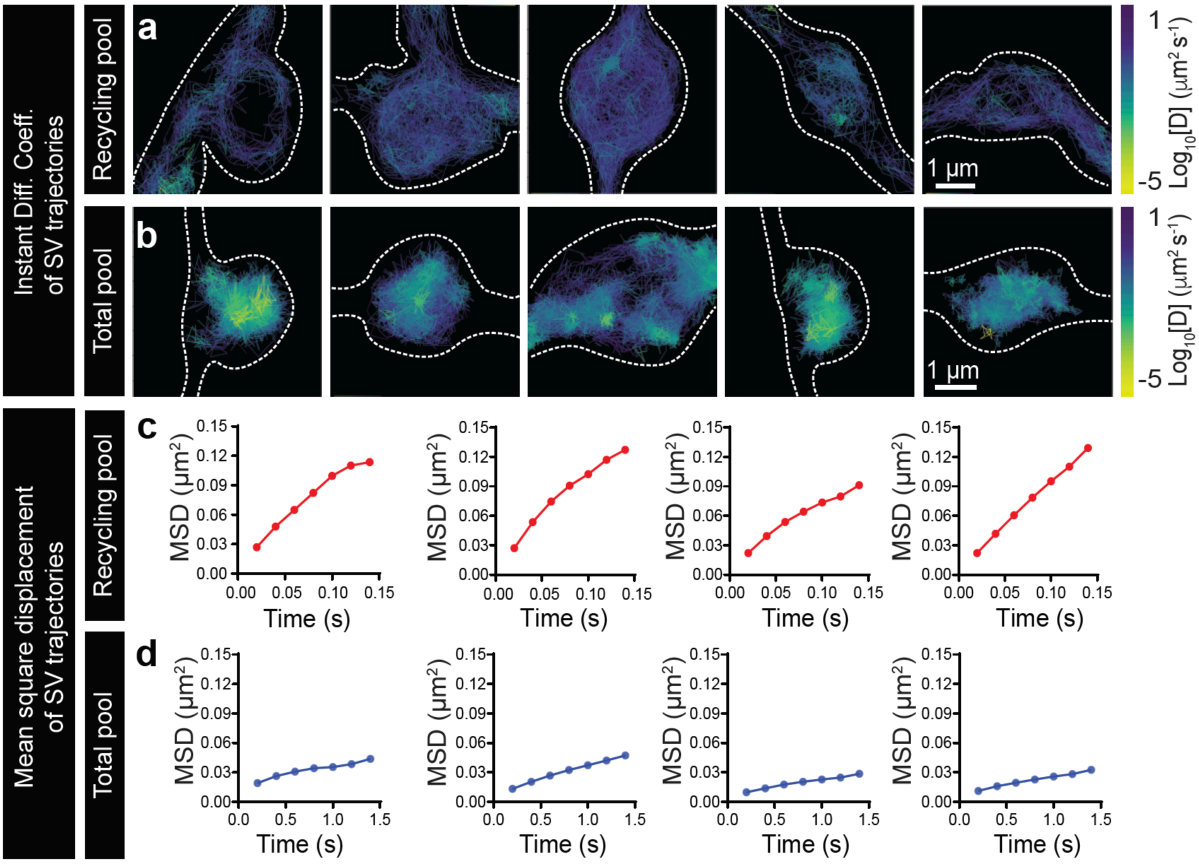

Supplementary Fig. S1. Examples of SV trajectories and their corresponding MSD curves.

a, Examples of instantaneous diffusion coefficient trajectory maps of SV-containing VAMP2-pHluorin tagged with anti-GFP At647N-GBP nanobodies (Recycling pool) in presynaptic regions. **b,** Examples of instantaneous diffusion coefficient trajectory maps of SV-containing vGLUT1-mEos2 (Total pool) in presynaptic regions. The trajectories in (**a**) and (**b**) were generated using segNASTIC. The color-code of the trajectories indicates their instantaneous diffusion coefficients. **c,** Examples of MSDs of the trajectories from SV-containing VAMP2-pHluorin/At647N-GBP nanobodies (Recycling pool) in presynaptic regions, as a function of time. **d,** Examples of MSDs of the trajectories from SV-containing vGLUT1-mEos2 (Total pool) in presynaptic regions, as a function of time. Source data are provided as a Source Data file.

Supplementary Fig. S2

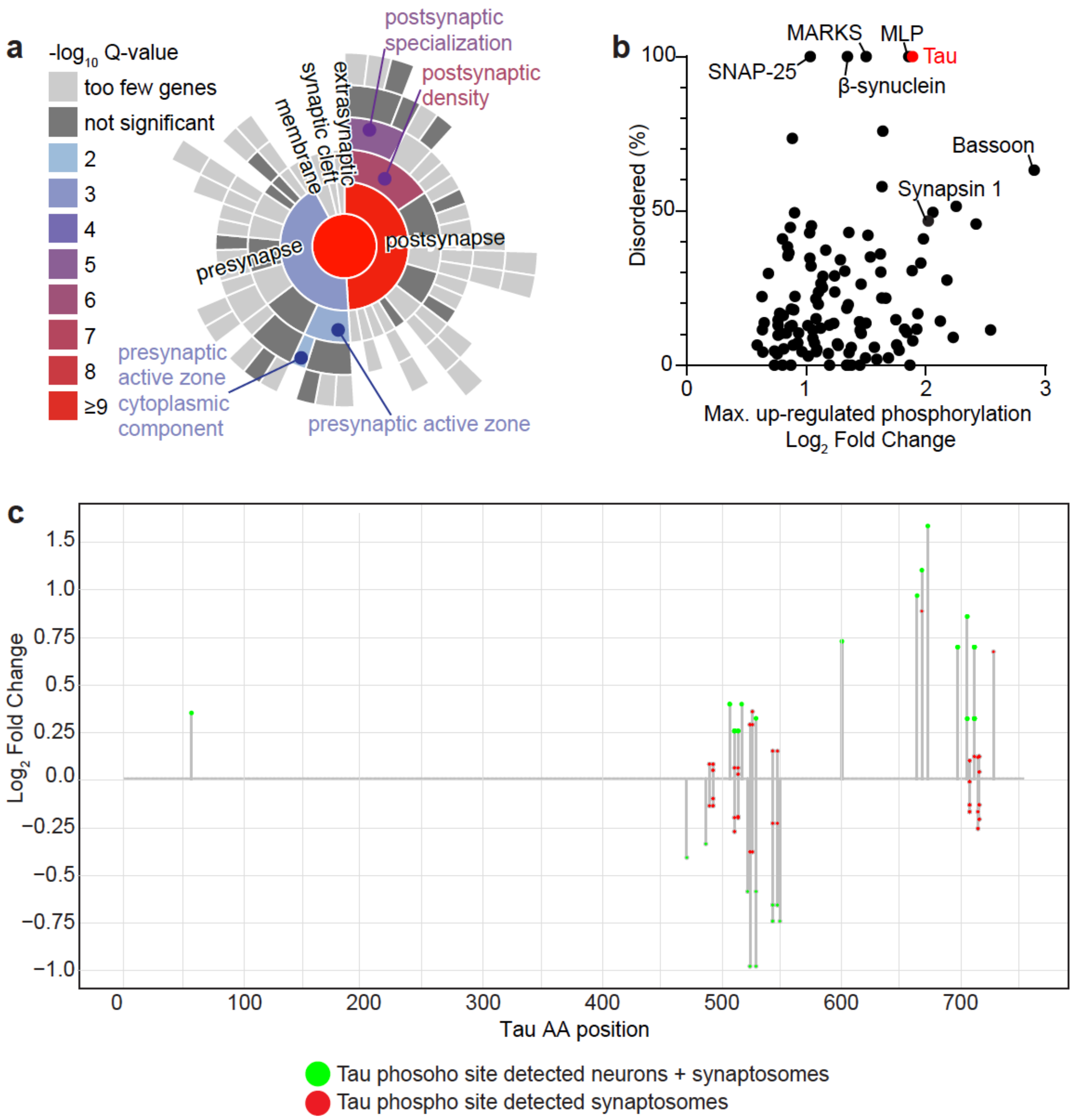

Supplementary Fig. S2. Tau is a high phosphorylated-high disordered candidate modulated by high K^+ stimulation.

a, SynGO (cellular component) analysis of the top 500 differentially phosphorylated proteins. Rat synaptosomes were stimulated with high K^+ or not stimulated (low K^+) for 10 s and the phosphoproteome was analyzed by mass spectrometry. The data from the rat synaptosome phosphoproteome [23] was then used to calculate a maximum log_2 (intensity) differential between high and low K^+ treatment for presynaptic proteins. **b**, Percentage disorder, provided by MobiDB [99] and DisPort [100] databases, was plotted against the maximum log_2 (intensity) differential. MLP, MARCKS-like protein 1. The data used to plot the graph is provided in

Supplementary Data 1. Although the concentration of K^+ and duration of stimulation differed between the rat and mouse phosphoproteomes, similar presynaptic proteins were among the most highly up-regulated proteins in the depolarized rat hippocampal neurons, and Tau was prominent in both preparations. **c**, Individual Tau phosphosites for mouse and rat, and their $\log_2$ intensities in the 10 s rat stimulation treatment. Source data are provided as a Source Data file and the Supplementary Data 1.

Supplementary Fig. S3

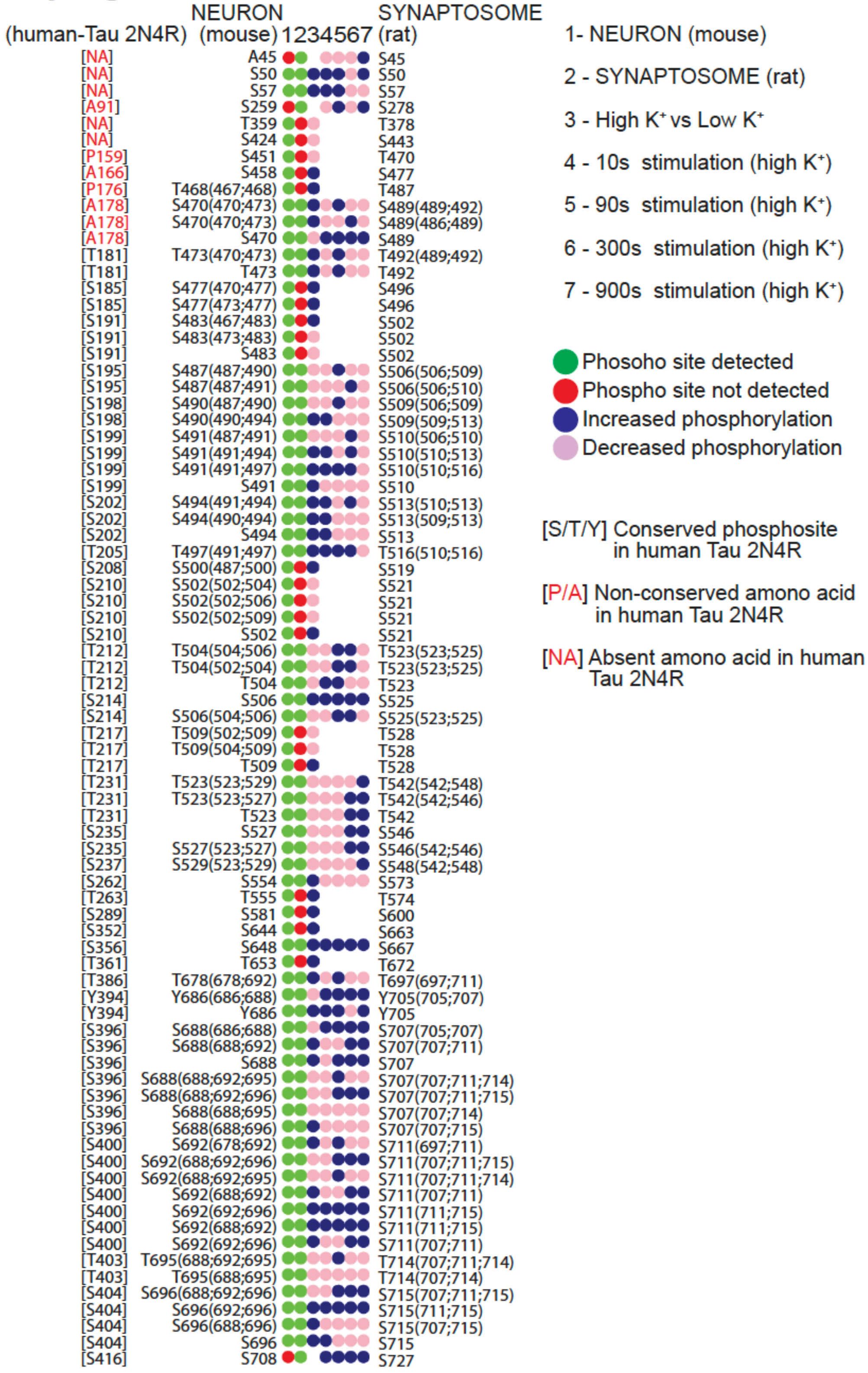

Supplementary Fig. S3. Tau activity-dependent phosphosites

Each neuronal (mouse) or synaptosomal (rat) identified Tau phosphosite, and the direction of phosphorylation in response to stimulation in low K^+ (mouse neurons) or after different

periods (10, 90, 300 or 900 seconds) of high K^+ stimulation (rat synaptosomes). Tau conserved and non-conserved residues in the human 2N4R isoform are also specified. Source data are provided as a Source Data file.

Supplementary Fig. S4

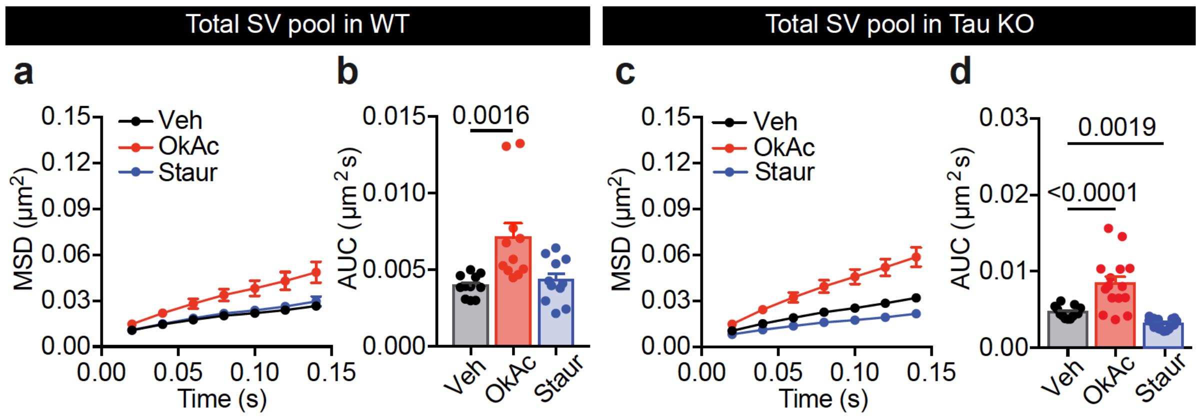

Supplementary Fig. S4. Effect of phosphorylation status on the mobility of the total pool of SVs in WT and Tau KO neurons.

a, Average MSD of the trajectories of total pool of SVs from neurons treated with control vehicle (Veh), okadaic acid (1 µM, 10 min, OkAc), or staurosporine (1 µM, 10 min, Staur) in WT neurons. **b,** The corresponding area under the MSD curves (AUC). **c,** Average MSD of the trajectories of total pool of SVs from neurons treated with control vehicle (Veh), okadaic acid (OkAc), or staurosporine (Staur) in Tau KO neurons. **d,** The corresponding area under the MSD curves (AUC). Data in (**b**) and (**d**) are displayed as mean ± SEM. Values were obtained from $n \geq 10$ neurons per condition, from > 2 independent neuronal cultures. Statistical comparisons in (**b**) and (**d**) were performed using the Kruskal-Wallis test and the Dunn's multiple comparisons test comparing the groups to the control (Veh). Source data are provided as a Source Data file.

Supplementary Fig. S5

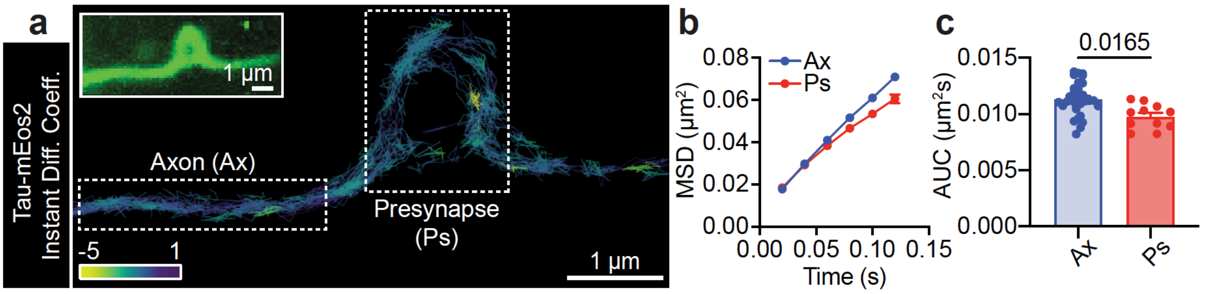

Supplementary Fig. S5. Synaptic Tau-mEos2 has lower mobility than axonal Tau-mEos2.

a, Representative example of the instantaneous diffusion coefficient trajectory map of Tau-mEos2. The trajectories were generated using segNASTIC. The color-code of the trajectories indicates their instantaneous diffusion coefficients (the color bar represents $\log_{10}[\mu m^2 s^{-1}]$). The inset (**i**) represents a lower magnification of the corresponding epifluorescent image of VAMP2-pHluorin. **b,** Average MSD of the trajectories of Tau-mEos2 molecules from axons and presynapses. **c,** The corresponding area under the curve (AUC). Data in (**b**) and (**c**) are displayed as mean ± SEM. Values were obtained from n ≥ 12 neurons per condition, from > 2 independent neuronal cultures. Statistical comparisons in (**c**) were performed using the Student's *t*-test with Welch's correction. Source data are provided as a Source Data file.

Supplementary Fig. S6

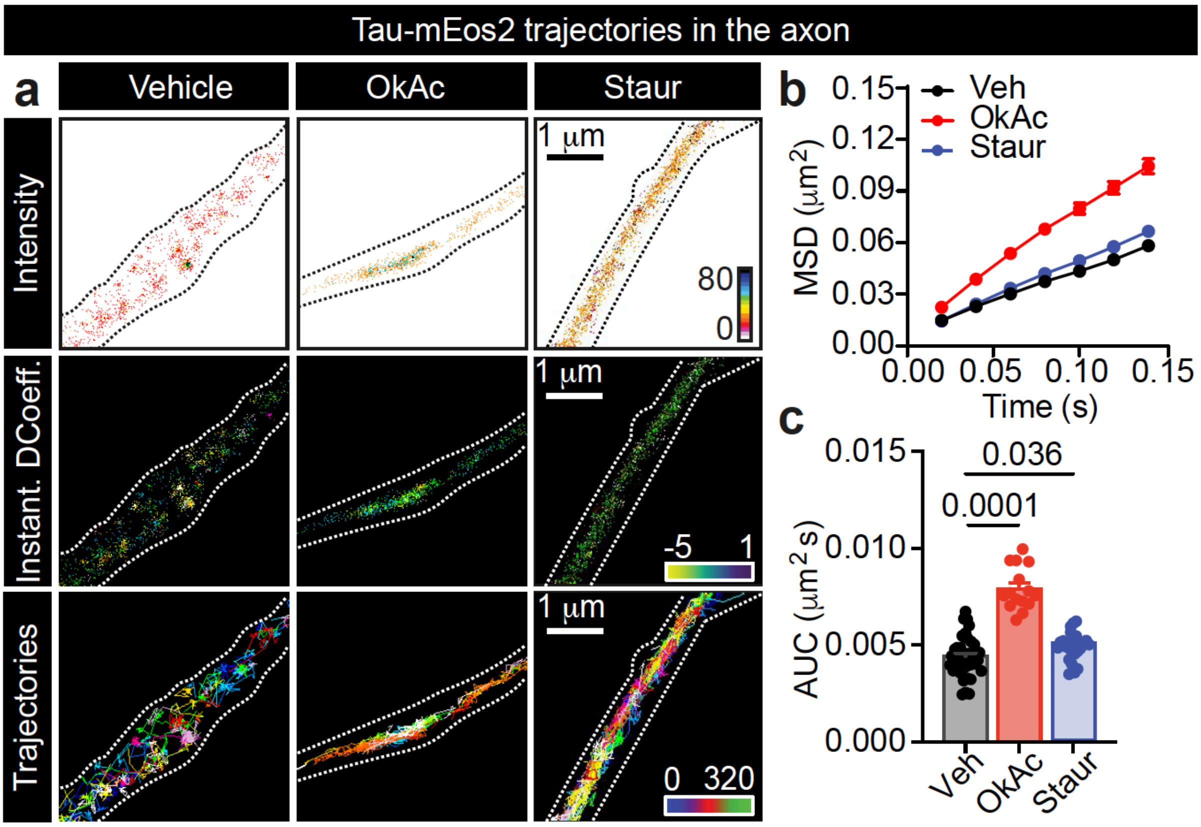

Supplementary Fig. S6. Okadaic acid-induced protein phosphorylation increases Tau-mEos2 mobility.

a, Representative images of axonal segments from WT neurons transfected with Tau-mEos2, treated with control vehicle (Veh), okadaic acid (OkAc) or staurosporine (Staur) during 15 min, as indicated. Plotting of the intensity map, diffusion coefficient map and trajectory map of Tau-mEos2 molecules, as indicated. **b,** Average MSD of the trajectories of Tau-mEos2 molecules from neurons in the indicated conditions. **c,** The corresponding area under the curve (AUC). Data in (**b**) and (**c**) are displayed as mean ± SEM. Values were obtained from n ≥ 15 neurons per condition, from > 3 independent neuronal cultures. Statistical comparisons in (**c**) were performed using the Brown-Forsythe and Welch ANOVA test followed by Dunnett t3 *post hoc* test comparing the groups to the control Veh. Source data are provided as a Source Data file.

Supplementary Fig. S7

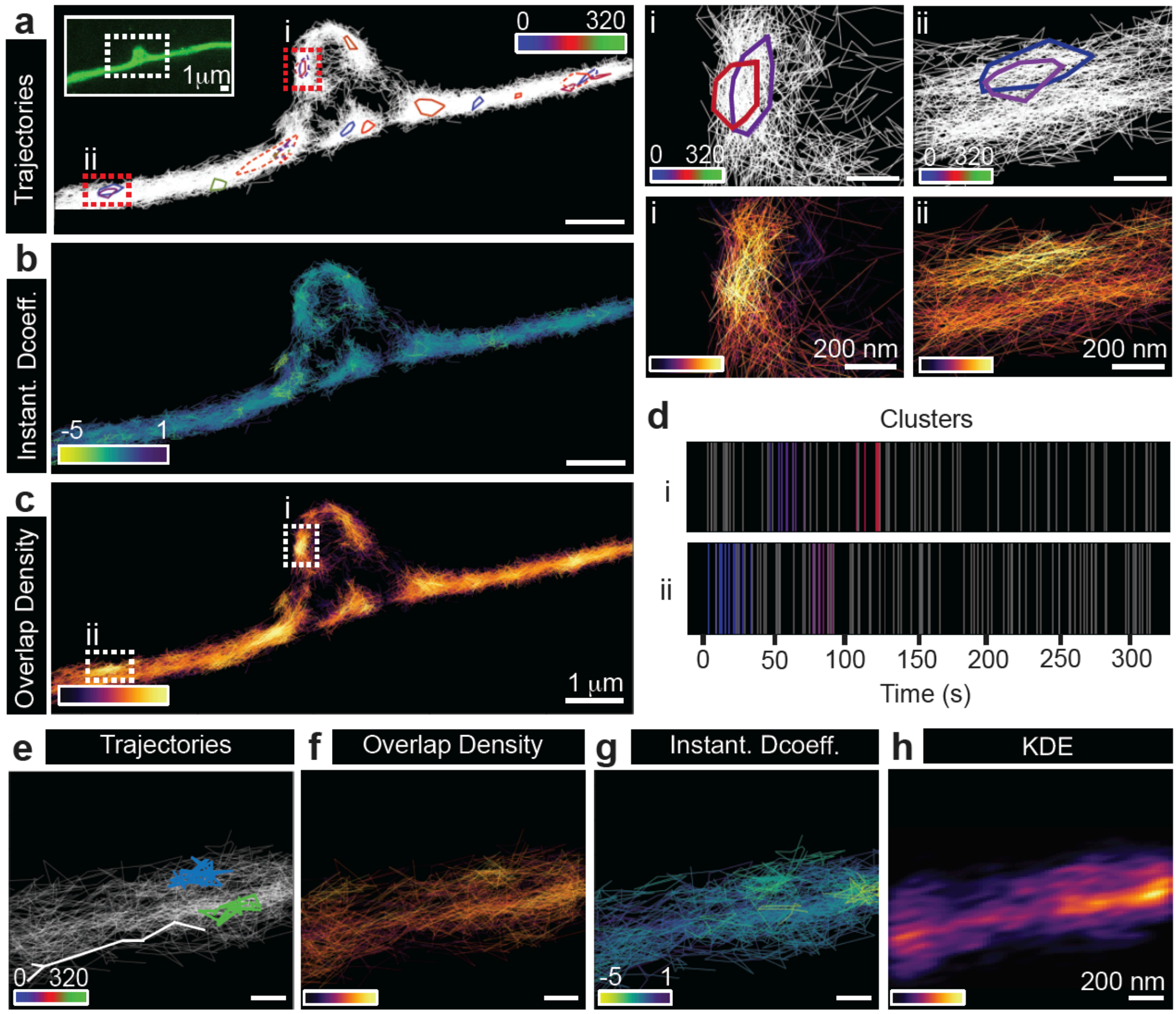

Supplementary Fig. S7. Tau-mEos2 organizes forming intermittent nanoclusters in axons and presynapses.

a, Representative Tau-mEos2 trajectories generated using segNASTIC showing nanoclusters (colored areas) at the presynapse and in the axonal compartment determined by VAMP2-pHluorin intensity (inset). Color-coding of the nanoclusters represents their appearance in time across the acquisition (16,000 frames, 320 s). The red insets in the presynaptic (**i**) and the axonal (**ii**) compartment are shown at a higher magnification. **b,** Representation of Tau-mEos2 trajectories generated using segNASTIC showing instantaneous diffusion coefficients. The color-code of the trajectories indicates their instantaneous diffusion coefficients (the color bar represents $\log_{10}[\mu m^2 s^{-1}]$). **c,** Representation of the overlap density of Tau-mEos2 trajectories. The color-code of the trajectories indicates their overlapping, with light colors given to the most overlapped trajectories. The red insets in the presynaptic (**i**) and the axonal (**ii**) compartment are shown at a higher magnification. **d,** Temporal representation of Tau-

mEos2 trajectories along the nanoclustered regions from the presynapse (**i**) or the axon (**ii**). The colors of the trajectories match with their belonging to specific clusters. **e,** Plotting of the trajectory map of Tau-mEos2 molecules highlighting examples of two clustered trajectories (blue and green color) and one non-clustered trajectory (white color). **f,** Plotting of the overlap density map of Tau-mEos2 molecules. **g,** Plotting of Tau-mEos2 trajectories showing instantaneous diffusion coefficients. **h,** Plotting of the 2D kernel density estimation of all directions (KDE). Source data are provided as a Source Data file.

Supplementary Fig. S8

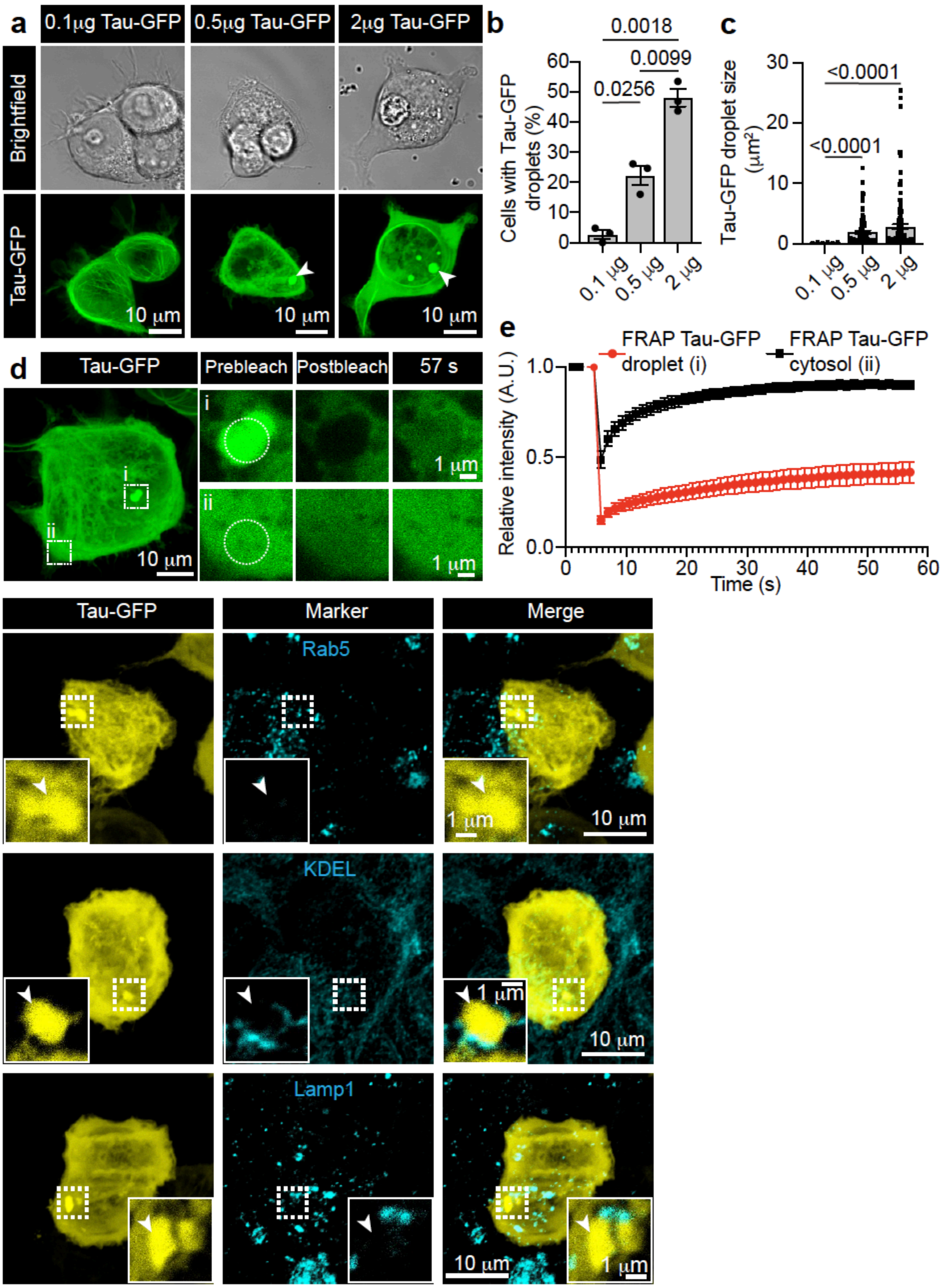

Supplementary Fig. S8. Tau-GFP forms biomolecular condensates in HEK-293T cells.

a, Representative images of HEK-293T cells transfected with increasing concentrations of Tau-GFP plasmid, as indicated. Arrowhead indicates the position of a Tau-GFP droplet. **b,**

Plot of the quantification of the percentage of cells containing Tau-GFP droplets. **c,** Plot of the quantification of the average size of Tau-GFP droplets. **d,** Representative images of HEK-293T cells expressing Tau-GFP, where FRAP analysis was carried out inside and outside Tau-GFP droplets. **e,** Plot of the FRAP analysis in the cytosol and the droplets, as indicated. **f-h,** Representative immunocytochemistry images of HEK-293T cells expressing Tau-GFP droplets. Immunocytochemistry of Tau and different membrane-bound organelle markers including (**f**) Rab5, (**g**) KDEL and (**h**) Lamp1. Data in (**b**), (**c**) and (**e**) are displayed as mean ± SEM. Statistical comparisons in (**b**) and (**c**) were performed using the Brown-Forsythe and Welch ANOVA test followed by Dunnett t3 *post hoc* test comparing the groups to the lowest concentration of Tau-GFP plasmid. Statistical comparisons were performed on a per-experiment basis in (**b**) (21 to 114 cells analyzed per experiment), or a per-cell basis in (**c**). Source data are provided as a Source Data file.

Supplementary Fig. S9

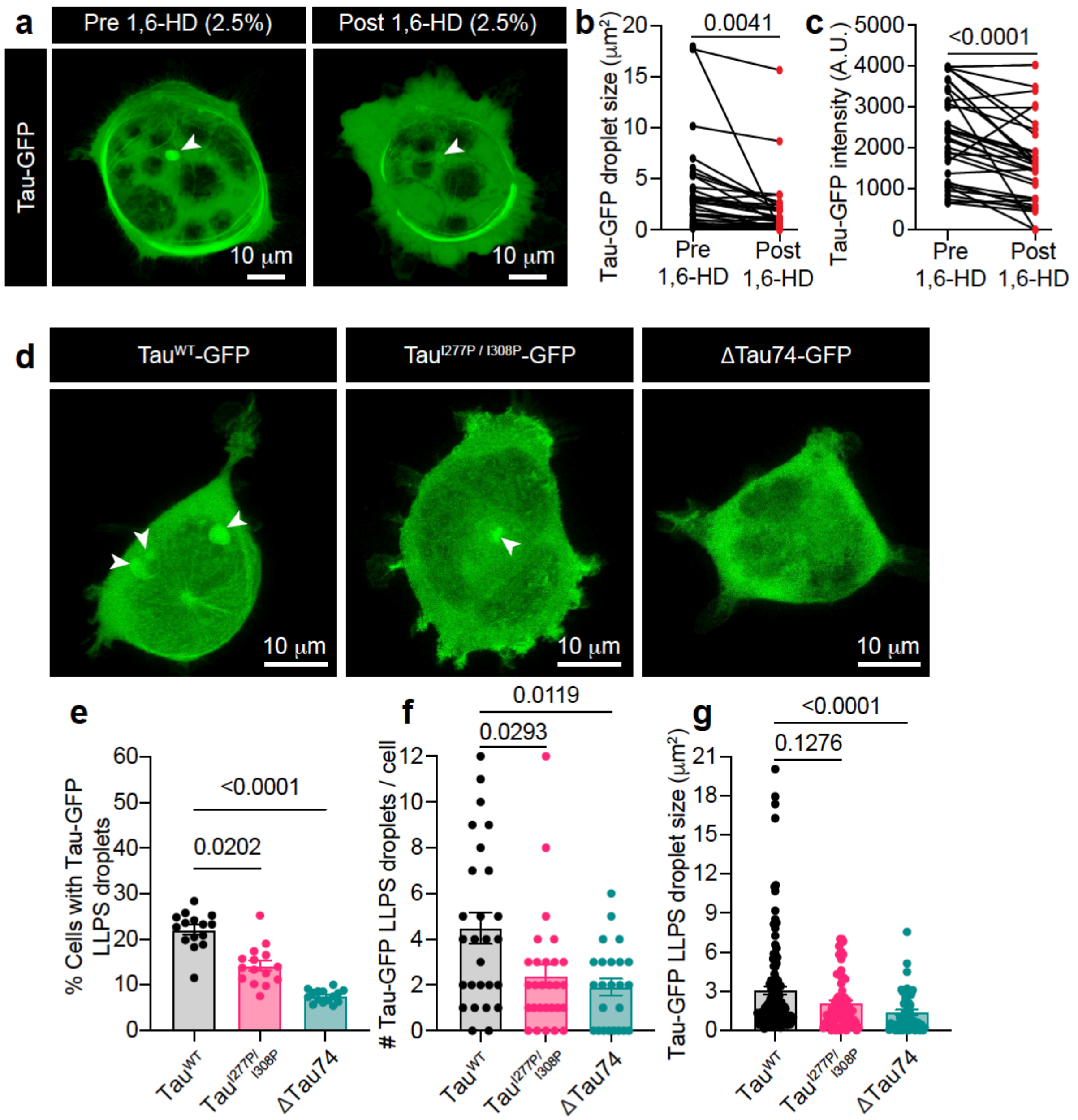

Supplementary Fig. S9. Tau condensates are sensitive to hexanediol.

a, Representative images of HEK-293T cells expressing Tau-GFP before and after treatment with 1,6-HD (2.5%). Arrowhead indicate the position of a Tau-GFP condensate. **b,** Plot of the size of Tau-GFP condensates before and after treatment with 1,6-HD. **c,** Plot of Tau-GFP fluorescence intensity in the condensates before and after treatment with 1,6-HD. **d,** Representative images of HEK-293T cells expressing Tau WT-GFP or the Tau LLPS mutants $Tau^{I277P / I308P}$-GFP and ΔTau74-GFP. **e,** Plot of the % of cells with Tau LLPS condensates. **f,** Plot of the number of Tau LLPS condensates per cell. **g,** Plot of the size of Tau-GFP condensates. Data in (**e**), (**f**) and (**g**) are displayed as mean ± SEM. 3 independent

cell cultures were performed per condition. Statistical comparisons in (**b**), (**c**) and (**f**) were performed on a per-cell basis. Statistical comparisons in (**g**) were performed on a per-droplet basis, and on a per-dish basis in (**e**). The Student's paired *t*-test was used in (**b**) and (**c**). One-way ANOVA Kruskal-Wallis test followed by Dunn's *post hoc* test comparing all groups to the Tau WT-GFP control was performed in (**e**), (**f**) and (**g**). Source data are provided as a Source Data file.

Supplementary Fig. S10

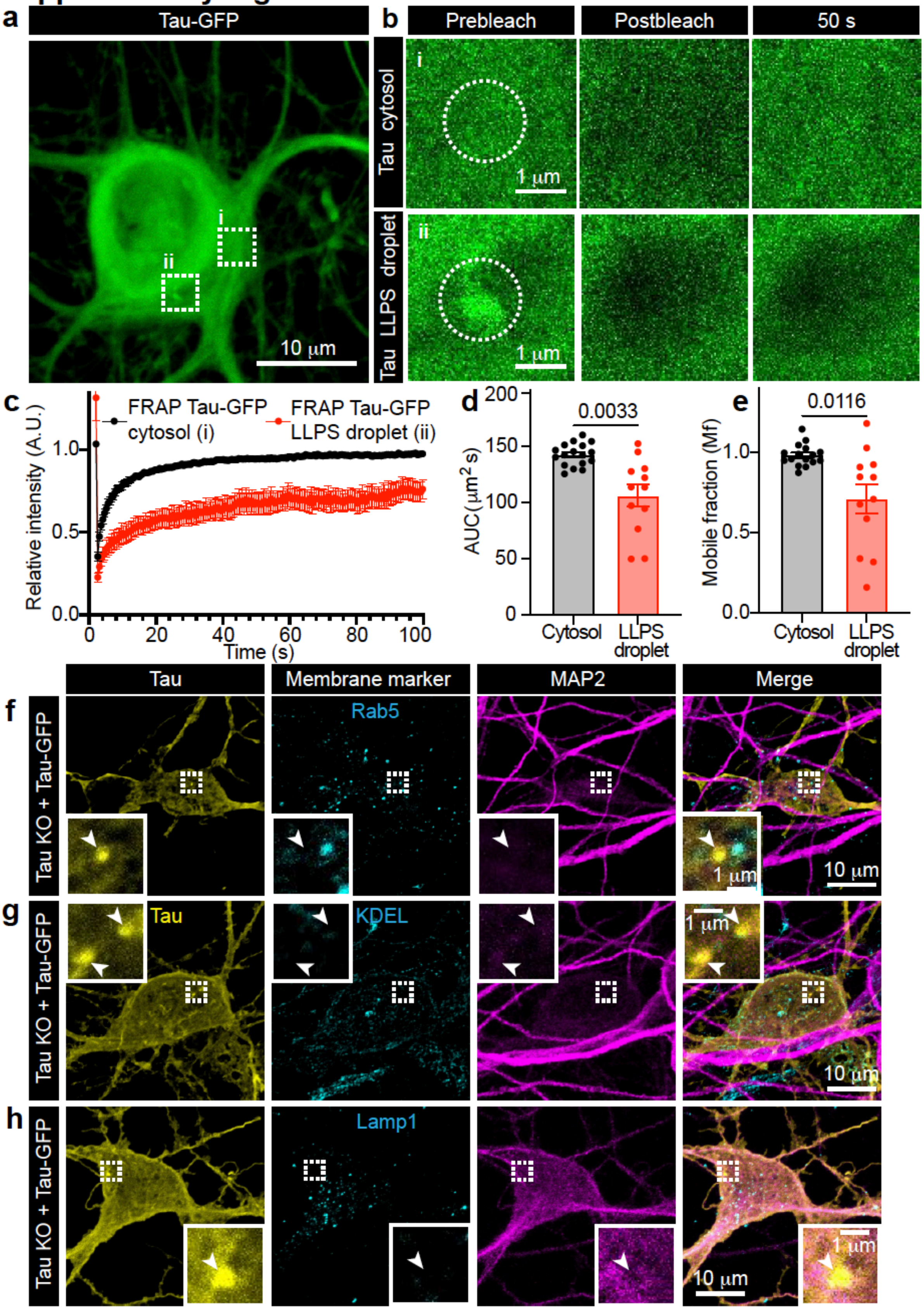

Supplementary Fig. S10. Tau-GFP forms biomolecular condensates in hippocampal neurons.

a, Representative image of a Tau KO hippocampal neuron expressing Tau-GFP, where FRAP analysis was performed outside (**i**) and inside (**ii**) Tau-GFP condensates (droplets). **b**, Representative regions taken during the FRAP experiments of Tau-GFP outside (**i**) and inside (**ii**) condensates. **c,** Plot of the FRAP analysis in the cytosol and the droplets, as indicated. **d**, Quantification of the area under the FRAP curve (AUC). **e,** Quantification of the mobile fraction of the FRAP curves. **f-h,** Representative immunocytochemistry images of Tau KO neurons expressing Tau-GFP droplets. Immunocytochemistry of Tau, MAP2 and indicated membrane-bound organelles markers, including (**f**) Rab5, (**g**) KDEL and (**h**) Lamp1. Data in (**d**) and (**e**) are displayed as mean ± SEM. Values were obtained from $n \geq 12$ neurons per condition from 2 independent neuronal cultures. Statistical comparisons in (**d**) and (**e**) were performed using the Student's paired *t*-test with Welch's correction. Source data are provided as a Source Data file.

Supplementary Fig. S11

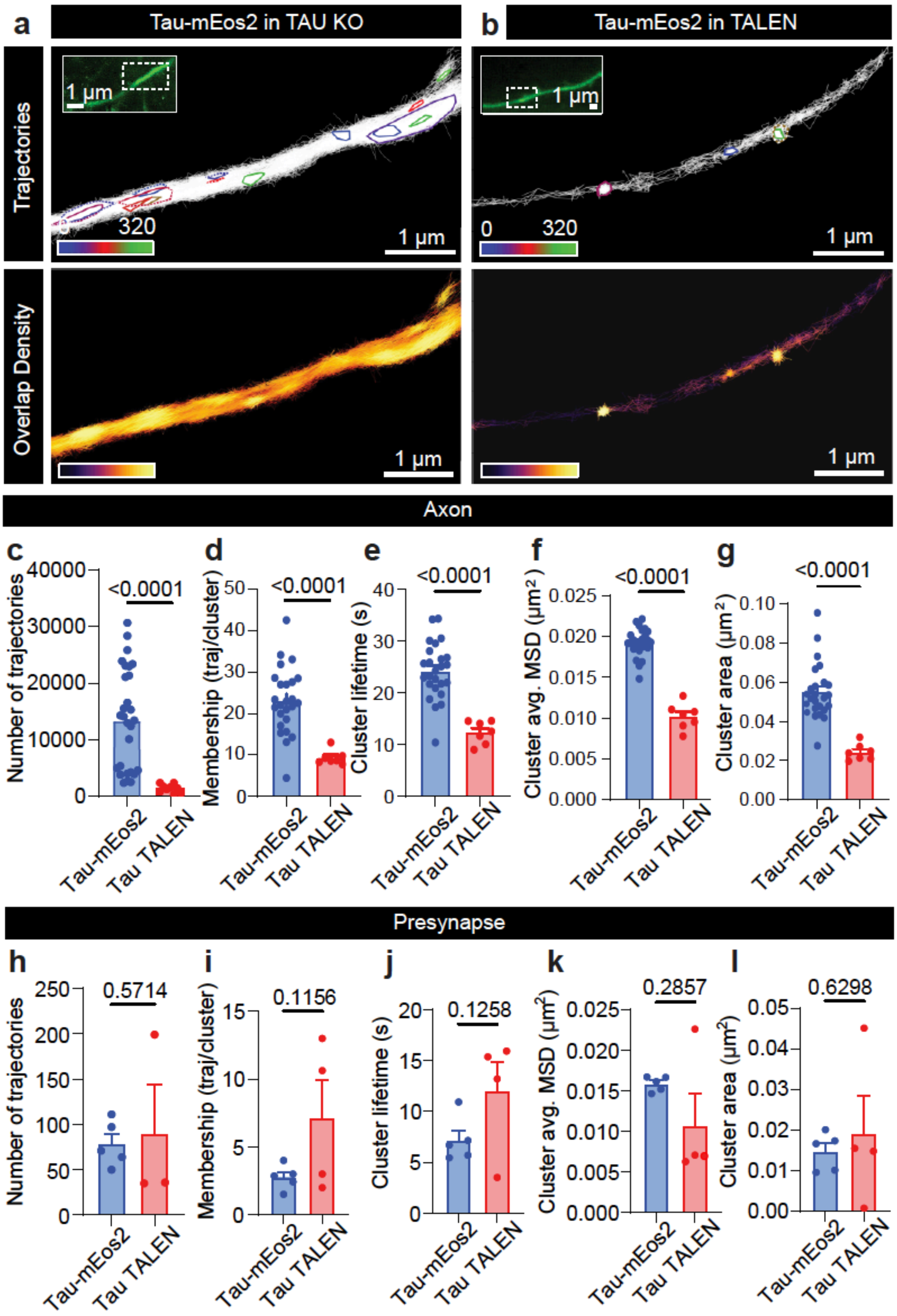

Supplementary Fig. S11. Tau-mEos2 protein concentration affects its nanocluster organization.

a, Rescued Tau-mEos2 expression in Tau KO hippocampal neurons. Representative Tau-mEos2 trajectories were generated using segNASTIC and nanoclusters are indicated as colored circular regions. Color-coding of the nanoclusters represents their appearance in time across the acquisition (16,000 frames, 320 s). The neuronal compartment determined by the presence of VAMP2-pHluorin (inset). **b,** Endogenous Tau molecules were imaged in TALEN gene edited Tau-mEos2 mice expressing low levels of Tau protein. Representative Tau-mEos2 trajectories were generated using segNASTIC and nanoclusters are indicated. Color-coding of the nanoclusters represents their appearance in time across the acquisition (16,000 frames, 320 s). The neuronal compartment is determined by the presence of VAMP2-pHluorin (inset). **c-l,** Metric comparison of clustered Tau-mEos2 trajectories in the axonal compartment (**c-g**) or presynapses (**h-l**) from re-expressed Tau-mEos2 (Tau-mEos2) neurons or endogenous Tau-mEos2 TALEN neurons. (**c, h**) Average number of Tau-mEos2 trajectories inside nanoclusters. (**d, i**) Average nanocluster membership (trajectories / cluster). (**e, j**) Average lifetime of Tau-mEos2 nanoclusters. (**f, k**) Average area under the curves (AUC) of the MSD curves of clustered Tau-mEos2 trajectories. (**g, l**) Average Tau-mEos2 nanocluster area. Data are displayed as mean ± SEM. Statistical comparisons were performed using the Student's *t*-test with Welch's correction. Source data are provided as a Source Data file.

Supplementary Fig. S12

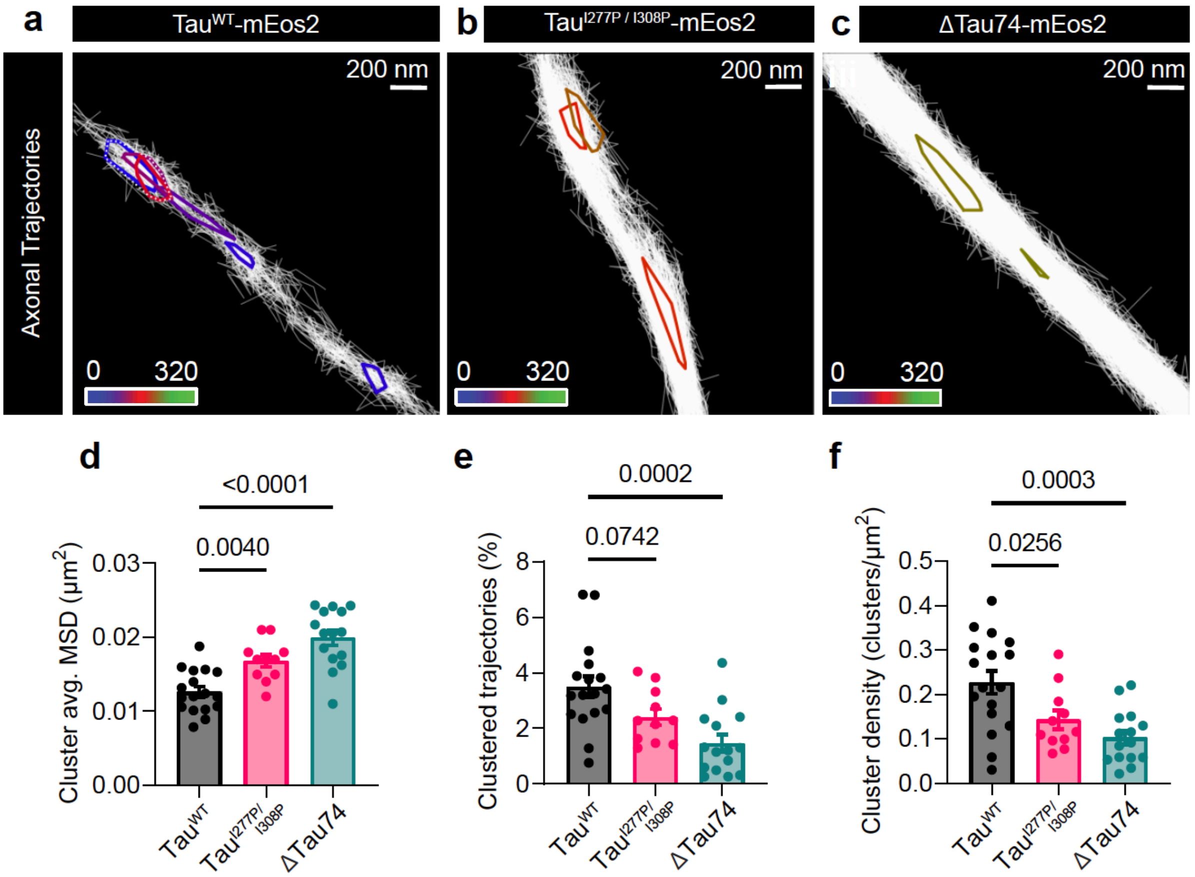

Supplementary Fig. S12. Impairing Tau LLPS affects tau nanoclusters in the axonal compartment.

a-c, Representative trajectories from (**a**) mEos2-tagged Tau WT, (**b**) $Tau^{I277P / I308P}$ and (**c**) ΔTau74. Images were generated using segNASTIC showing nanoclusters in the axonal compartment. Color-coding of the clusters represents their appearance in time across the acquisition (16,000 frames, 320 s). **d**, Average MSD of clustered mEos2-tagged Tau WT and Tau LLPS mutants' trajectories represented as the area under the curves (AUC). **e**, Percentage clustered trajectories. **f**, Cluster density represented as the number of clusters per μm^2. Data in (**d-f**) are displayed as mean ± SEM. Data was obtained from 2 independent neuronal cultures. Statistical comparisons were performed using the one-way ANOVA test. Source data are provided as a Source Data file.

Supplementary Fig. 13

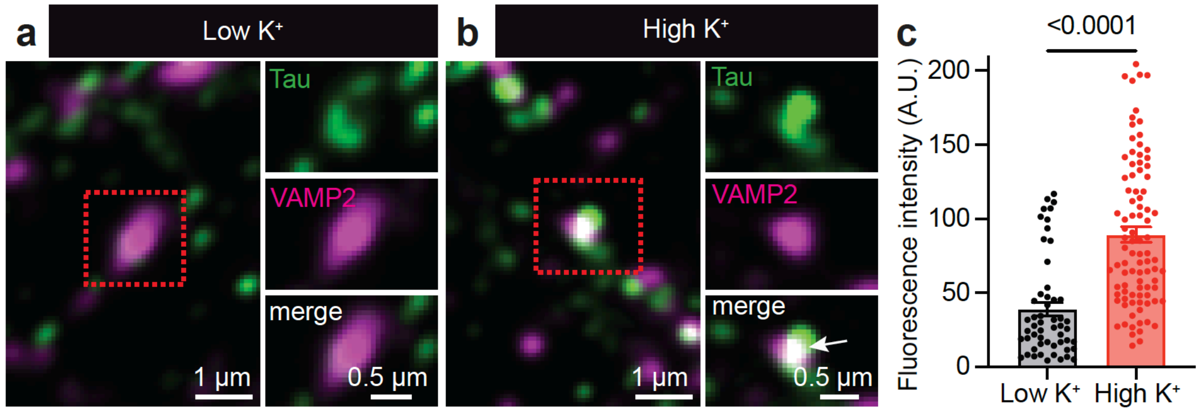

Supplementary Fig. S13. High K$^+$ stimulation induces the synaptic relocalization of Tau.

a, b, Representative images of WT neurons in (**a**) resting conditions (low K$^+$) and (**b**) stimulated conditions (high K$^+$), stained with anti-Tau-5 and anti-VAMP2. Inset represents a presynaptic region. **c,** Plot of anti-Tau-5 fluorescence intensity signal that colocalizes with anti-VAMP2 signal. Data in (**c**) are displayed as mean ± SEM. Statistical comparison was performed using the Mann-Whitney U-test. Source data are provided as a Source Data file.
