# Supplementary material for: Tau forms synaptic nano-biomolecular condensates controlling the dynamic clustering of recycling synaptic vesicles
Source: Nat Commun. 2023 Nov 10;14:7277. doi: 10.1038/s41467-023-43130-4 (PMC10638352; doi:10.1038/s41467-023-43130-4)
Supplement: Supplementary file 3 — Description of Additional Supplementary Files [file 41467_2023_43130_MOESM3_ESM.pdf]

Description of Additional Supplementary Files

File Name: Supplementary Data 1
Description: Candidate proteins identified from the mouse hippocampus phosphoproteome and rat synaptosome analyses based on the maximum upregulated phosphorylation value (elicited by simulation) and the percentage of disordered regions.

File Name: Supplementary Movie S1
Description: SdTIM imaging. Representative movie of the image acquisition during an sdTIM experiment to visualize the mobility and organization of recycling SVs. Recycling SVs are tagged with anti-GFP nanobodies tagged with Atto647 bound to VAMP2-pHluorin. 16,000 images are acquired every 20 ms.

File Name: Supplementary Movie S2
Description: SptPALM imaging. Representative movie of the image acquisition during an sptPALM experiment to visualize the mobility and organization of the total pool of SVs. The total pool of SVs is tagged by the presence of vGLUT1- mEos2. 16,000 images are acquired every 20 ms.

File Name: Supplementary Movie S3
Description: Dual-color sdTIM/sptPALM imaging. Representative movie of the image acquisition during a dual-color sdTIM/sptPALM experiment to simultaneously visualize the mobility and organization of recycling SVs (sdTIM) and Tau-mEos2 molecules (sptPALM). Recycling SVs are tagged with anti-GFP nanobodies tagged with Atto-647 bound to VAMP2-pHluorin. 16,000 images are acquired every 20 ms.
