# Supplementary material for: Tau forms synaptic nano-biomolecular condensates controlling the dynamic clustering of recycling synaptic vesicles
Source: Nat Commun. 2023 Nov 10;14:7277. doi: 10.1038/s41467-023-43130-4 (PMC10638352; doi:10.1038/s41467-023-43130-4)
Supplement: Supplementary file 8 — Reporting Summary [file 41467_2023_43130_MOESM8_ESM.pdf]

nature portfolio

Corresponding author(s): Ramon Martinez-Marmol
Frederic A. Meunier

Last updated by author(s): Oct 15 2023

Reporting Summary

Nature Portfolio wishes to improve the reproducibility of the work that we publish. This form provides structure for consistency and transparency in reporting. For further information on Nature Portfolio policies, see our Editorial Policies and the Editorial Policy Checklist.

Statistics

For all statistical analyses, confirm that the following items are present in the figure legend, table legend, main text, or Methods section.

n/a	Confirmed	
☐	☒	The exact sample size (*n*) for each experimental group/condition, given as a discrete number and unit of measurement
☐	☒	A statement on whether measurements were taken from distinct samples or whether the same sample was measured repeatedly
☐	☒	The statistical test(s) used AND whether they are one- or two-sided<br>*Only common tests should be described solely by name; describe more complex techniques in the Methods section.*
☒	☐	A description of all covariates tested
☐	☒	A description of any assumptions or corrections, such as tests of normality and adjustment for multiple comparisons
☐	☒	A full description of the statistical parameters including central tendency (e.g. means) or other basic estimates (e.g. regression coefficient) AND variation (e.g. standard deviation) or associated estimates of uncertainty (e.g. confidence intervals)
☐	☒	For null hypothesis testing, the test statistic (e.g. *F*, *t*, *r*) with confidence intervals, effect sizes, degrees of freedom and *P* value noted<br>*Give P values as exact values whenever suitable.*
☐	☒	For Bayesian analysis, information on the choice of priors and Markov chain Monte Carlo settings
☒	☐	For hierarchical and complex designs, identification of the appropriate level for tests and full reporting of outcomes
☐	☒	Estimates of effect sizes (e.g. Cohen's *d*, Pearson's *r*), indicating how they were calculated

Our web collection on statistics for biologists contains articles on many of the points above.

Software and code

Policy information about availability of computer code

Data collection	Confocal images were acquired using Zeiss Zen Black software (Zeiss) or SlideBook 6.0 software (31, Inc), as detailed in the method section. Super-resolution imaging was acquired using Metamorph software (version 7.7.8, Molecular Devices, CA, USA), as detailed in the method section. Mass Spectrometry data was collected using Xcalibur v4.2.57 (Thermo Scientific).
Data analysis	FIJI-Image J (version 2.0.0-rc-04Tll:30:09+0000), MetaMorph (Molecular Devices), PALM-Tracer (version 2.1.0.28228), MATLAB (MathWorks, Inc.), segNASTIC software (v20220221), GraphPadPrism software (Graph Pad Software, Inc), Microsoft Excel (version 16.62). MaxQuant (version 1.6.7.0). Computer codes to analyze the data have been described in the work by Wallis et al 2023 Nature Communications. Full details about how the code can be accessed is in that paper.

For manuscripts utilizing custom algorithms or software that are central to the research but not yet described in published literature, software must be made available to editors and reviewers. We strongly encourage code deposition in a community repository (e.g. GitHub). See the Nature Portfolio guidelines for submitting code & software for further information.

Data

Policy information about availability of data

All manuscripts must include a data availability statement. This statement should provide the following information, where applicable:

- Accession codes, unique identifiers, or web links for publicly available datasets
- A description of any restrictions on data availability
- For clinical datasets or third party data, please ensure that the statement adheres to our policy

The accession code of the datasets deposited to ProteomeXchange Consortium via the PRIDE are PXD020232 and 10.6019/PXD020232. They are also available in manuscript.
The MobiDB (https://mobidb.bio.unipd.it/)and DisProt (https://disprot.org/) databases were used to experimentally determined percentage of intrinsically disordered sequence for each presynaptic protein. The Mus musculus and Rattus norvegicus data from MobiDB, and the Mus musculus, Rattus norvegicus and Homo sapiens data from DisProt were download to obtain the values for the predicted percentage of disordered sequence content. The stimulated data from the manuscript Engholm-Keller et al 44 was used to align each phosphorylated protein to its homologous Rattus norvegicus in the experiment of neuronal stimulation with high K+.
Single-particle trajectory data generated in this study are available for download from the publicly accessible institutional data repository of The University of Queensland (UQ eSpace) 10.48610/a9c9def.
Further information and request for reagents and resources should be directed to the corresponding authors.
Source data are provided with this paper.

Research involving human participants, their data, or biological material

Policy information about studies with human participants or human data. See also policy information about sex, gender (identity/presentation), and sexual orientation and race, ethnicity and racism.

Reporting on sex and gender	N/A
Reporting on race, ethnicity, or other socially relevant groupings	N/A
Population characteristics	N/A
Recruitment	N/A
Ethics oversight	N/A

Note that full information on the approval of the study protocol must also be provided in the manuscript.

Field-specific reporting

Please select the one below that is the best fit for your research. If you are not sure, read the appropriate sections before making your selection.

[x] Life sciences [ ] Behavioural & social sciences [ ] Ecological, evolutionary & environmental sciences

For a reference copy of the document with all sections, see nature.com/documents/nr-reporting-summary-flat.pdf

Life sciences study design

All studies must disclose on these points even when the disclosure is negative.

Sample size	No statistical methods were used to predetermine sample sizes because our sample sizes were estimated based on those reported in similar previous publications (Joensuu et al. J Cell Biol. 2016 Oct 24;215(2):277-292; Padmanabhan et al., 2019, eLife 8:e45040; Martinez-Marmol et al., Mol Psychiatry. 2023 Feb;28(2):946-962). All the results for each condition are obtained from 2 to 4 independent neuronal dissections, pooling over 5 embryos per dissection. Respective n values are shown in figure captions. Results using cell lines were obtained from 3 independent experiments. Respective n values are shown in figure captions.
Data exclusions	Outliers were automatically identified based on their position at over two standard deviations from the mean, using the custom-made Python script Outlier Wrangler (Wallis, T.P., et al. bioRxiv, 2021.2009.2008.459552 (2021)). Outliers were automatically excluded from the dataset, as stated in the "Statistical analysis" section from the "Methods". In the mass spectrometry and proteomic experiments, single-charged ions and those with charges > 8 were excluded, and phosphopeptides that were not significantly regulated in the high K+ condition versus low K+ were excluded. Exclusion criteria was pre-established.
Replication	Neurons were collected from at least two independent experiments, and each experiment was derived from pooling neurons from over 5 dissected embryos. Results using cell lines were obtained from 3 independent experiments. All replication attempts were successful.
Randomization	Analyzed neurons and cells used for imaging were selected randomly from the culture dishes. Neurons were obtained from mixing the brains of over 5 dissected embryos (numbers vary depending on the pregnancies, as all embryos from the same pregnant mouse were used). The same number of dissected neurons were plated into dishes. Plating is considered random as it is impossible to know the origin of each neuron plated (to what embryo correspond). The pool of dishes generated are mixed and randomly divided into different treatments. The assignment

of treatments is performed by simple randomization using Excel.

Blinding	Our experiments were not performed blind. However, the data within the experiments was collected and re-analyzed independently by different researchers. We also used computer-based single-molecule automatic detection and tracking to minimize bias. Detection, tracking and analysis conditions were maintained constant between experimental conditions.

Reporting for specific materials, systems and methods

We require information from authors about some types of materials, experimental systems and methods used in many studies. Here, indicate whether each material, system or method listed is relevant to your study. If you are not sure if a list item applies to your research, read the appropriate section before selecting a response.

Materials & experimental systems

n/a	Involved in the study
☐	☒ Antibodies
☐	☒ Eukaryotic cell lines
☒	☐ Palaeontology and archaeology
☐	☒ Animals and other organisms
☒	☐ Clinical data
☒	☐ Dual use research of concern
☒	☐ Plants

Methods

n/a	Involved in the study
☒	☐ ChIP-seq
☒	☐ Flow cytometry
☒	☐ MRI-based neuroimaging

Antibodies

Antibodies used	Mouse monoclonal Anti-VAMP2 (1:500, Synaptic Systems, #104 211).<br>Mouse monoclonal Anti-Tau clone 5 (1:1000, Sigma-Aldrich, #MABN162).<br>Guinea pig polyclonal anti-MAP2 (1:200, Synaptic Systems, #188004).<br>Anti-GFP nanobodies tagged with Atto-647 (100 pM, Synaptic Systems, #N0301-AF647-L).<br>Anti-GFP chicken polyclonal antibody (1:1000, MERK, #AB16901).<br>Anti-Rab5 rabbit monoclonal antibody (1:1000, Abcam #ab218624).<br>Anti-KDEL rabbit monoclonal antibody (1:200, Abcam, #ab176333).<br>Anti-Lamp1 mouse monoclonal antibody (1:100, Abcam, #ab25630).<br>Anti-Tau mouse monoclonal antibody T46 (1:100, ThermoFisher, #13-6400).<br>Alexa Fluor 488 conjugated anti-mouse lgG (1:500, Thermo Fisher Scientific; #A32723); Alexa Fluor 488 conjugated anti-chicken lgG (1:500, Thermo Fisher Scientific; #A-11039), Alexa Fluor 546 conjugated anti-guinea pig lgG (1:500, Thermo Fisher Scientific; #A11074); Alexa Fluor 555 conjugated anti-rabbit lgG (1:500, Thermo Fisher Scientific; #A32732), Alexa Fluor 555 conjugated anti-mouse lgG (1:500, Thermo Fisher Scientific; #A32727), Alexa Fluor 546 conjugated anti-chiken lgG (1:500, Thermo Fisher Scientific; #A11040), Alexa Fluor 647 conjugated anti-rabbit lgG (1:500, Thermo Fisher Scientific; #A-31573), Alexa Fluor 647 conjugated anti-guinea pig lgG (1:500, Thermo Fisher Scientific; #A-21450)
Validation	We did not validate the specificity of any antibody in house. All used antibodies are validated by the manufacturers as inficated on the manufacturers's website (see below):<br>- Anti-VAMP2 (https://www.sysy.com/product/104211).<br>- Anti-Tau clone 5 (https://www.sigmaaldrich.com/AU/en/product/mm/mabn162).<br>- Anti-MAP2 (https://sysy.com/product/188004).<br>- Anti-Rab5 (https://www.abcam.com/products/primary-antibodies/rab5-antibody-epr21801-early-endosome-marker-ab218624.html).<br>- Anti-KDEL (https://www.abcam.com/products/primary-antibodies/kdel-antibody-epr12668-ab176333.html).<br>- Anti-Lamp1 (https://www.abcam.com/products/primary-antibodies/lamp1-antibody-h4a3-ab25630.html).<br>- Anti-Tau mouse T46 (https://www.thermofisher.com/antibody/product/Tau-Antibody-clone-T46-Monoclonal/13-6400).<br>- Anti-GFP (https://www.merckmillipore.com/AU/en/product/Anti-Green-Fluorescent-Protein-Antibody,MM_NF-AB16901).<br>- Alexa Fluor" 488 conjugated anti-mouse lgG (https://www.thermofisher.com/antibody/product/Goat-anti-Mouse-IgG-H-L-Highly-Cross-Adsorbed-Secondary-Antibody-Polyclonal/A32723).<br>- Alexa Fluor" 488 conjugated anti-chicken lgG (https://www.thermofisher.com/antibody/product/Goat-anti-Chicken-IgY-H-L-Secondary-Antibody-Polyclonal/A-11039).<br>- Alexa Fluor" 546 conjugated anti-guinea pig lgG (https://www.thermofisher.com/antibody/product/Goat-anti-Guinea-Pig-IgG-H-L-Secondary-Antibody-Polyclonal/A-11074).<br>- Alexa Fluor" 555 conjugated anti-rabbit lgG (https://www.thermofisher.com/antibody/product/Goat-anti-Rabbit-IgG-H-L-Highly-Cross-Adsorbed-Secondary-Antibody-Polyclonal/A32732).<br>- Alexa Fluor 555 conjugated anti-mouse lgG (https://www.thermofisher.com/antibody/product/Goat-anti-Mouse-IgG-H-L-Highly-Cross-Adsorbed-Secondary-Antibody-Polyclonal/A32727).<br>- Alexa Fluor 546 conjugated anti-chiken lgG (https://www.thermofisher.com/antibody/product/Goat-anti-Chicken-IgY-H-L-Secondary-Antibody-Polyclonal/A-11040).<br>- Alexa Fluor" 647 conjugated anti-rabbit (https://www.thermofisher.com/antibody/product/Donkey-anti-Rabbit-IgG-H-L-Highly-Cross-Adsorbed-Secondary-Antibody-Polyclonal/A-31573).<br>- Alexa Fluor 647 conjugated anti-guinea pig lgG (https://www.thermofisher.com/antibody/product/Goat-anti-Guinea-Pig-IgG-H-L-

Highly-Cross-Adsorbed-Secondary-Antibody-Polyclonal/A-21450).

Eukaryotic cell lines

Policy information about cell lines and Sex and Gender in Research

Cell line source(s)	HEK-293T cells (293T/17 [HEK 293T/17] (ATCC® CRL11268™))
Authentication	This cell line was not authenticated in house for this manuscript. The cell line used has been authenticated by the manufacturer (https://www.atcc.org/products/crl-11268). Short Tandem Repeat (STR) profiling report for Amelogenin: X, CSF1PO: 11, 12, D13S317: 12, 14, D16S539: 9, 13, D5S818: 8, 9, D7S820: 11, THO1: 7, 9.3, TPOX: 11, vWA: 16, 18, 19.
Mycoplasma contamination	The cells used in this study are regularly tested for Mycoplasma contamination and verified as Mycoplasma free.
Commonly misidentified lines (See ICLAC register)	The cell line used is not a misidentified line.

Animals and other research organisms

Policy information about studies involving animals; ARRIVE guidelines recommended for reporting animal research, and Sex and Gender in Research

Laboratory animals

-Wild type mice: C57BL/6 strain
-Tau knock-out (KO) mice strain (Dawson, H.N., et al. Inhibition of neuronal maturation in primary hippocampal neurons from tau deficient mice. J Cell Sci 114, 1179-1187 (2001)).
-TALEN gene-edited Tau-mEos2 mice strain (Xia, D., Gutmann, J.M. & Gotz, J. Mobility and subcellular localization of endogenous, gene-edited Tau differs from that of over-expressed human wild-type and P301L mutant Tau. Sci Rep 6, 29074 (2016)).

Age adults used for mating: 5-12 weeks old. Experiments were performed in neurons isolated from embryos (no sex differentiation).

For all mice used:
- Cage/tank/housing system (type and dimensions): OptiMice standard caging system: 34.3cm L, 29.2cm W, 15.5cm H.
- Cage floor area: 484cm².
- Food: Mouse cubes (SF00-100)(Autoclaved).
- Bedding: Pura Chip Aspen Fine Sani Chips- Biological Associates (Autoclaved).
- Nesting material: Tissue and Enviro-Dri (Autoclaved).
- Additional nesting/home cage enrichment: Red or yellow house/tunnel or cardboard house/tunnel.
- Temperature and humidity parametres: 18°c - 24°c (30%-70% RH).
- Lighting (type, schedule and intensity): 12 L : 12 D 80% intensity.

Wild animals	No wild animals were used in the study.
Reporting on sex	Neurons were obtained from hippocampi derived from embryos at E16-17. At this stage, the sex is not identified, but as the male/female ratio is approximately 50%, we assume that our data are obtained from equal numbers of male- and female-derived neurons.
Field-collected samples	No field collected samples were used in the study.
Ethics oversight	All experimental procedures using animals were conducted under the guidelines of the Australian Code of Practice for the Care and Use of Animals for Scientific purposes and were approved by the University of Queensland Animal Ethics Committee (2016/AE000254; 2020/AE000439; 2020/AE000204).

Note that full information on the approval of the study protocol must also be provided in the manuscript.

Plants

Seed stocks	N/A
Novel plant genotypes	N/A
Authentication	N/A
